# Editorial

**Published:** 1865-01

**Authors:** 


### Editorial.

Mortality of Chicago for Jan. 1865.—The following table shows the mortality of Chicago for the month of January, 1865, as condensed from the Health Officer's report:—

| | | | |
|---|---|---|---|
| Accidents and Injuries, | 6 | Epilepsy, | 1 |
| Apoplexy or Congestion of Brain | 2 | Disease of Heart, | 1 |
| Inflammation of Brain, | 7 | Marasmus, | 4 |
| Inflammation of Lungs, | 9 | Burned, | 3 |
| Inflammation of Stomach & Bowels | 5 | Typhoid Fever, | 12 |
| Pulmonary Consumption, | 35 | Typhus Fever, | 1 |
| Disease of the Liver, | 1 | Paralysis, | 1 |
| Laryngitis or Croup, | 23 | Scarlet Fever, | 8 |
| Convulsions, | 18 | Small-Pox, | 10 |
| Childbed Fever, | 10 | Spinal Meningitis, | 1 |
| Diphtheria, | 5 | Sciatica, | 1 |
| Dropsy, | 10 | Old Age, | 4 |
| Hydrocephalus, | 6 | Stillborn, | 10 |
| Dysentery, | 1 | Unknown, | 21 |
| Diarrhœa, | 7 | | |
| Erysipelas, | 2 | Total, | 225 |

Ages of the Deceased.—Under 5 years, 100; over 5 and under 10 years, 14; over 10 and under 20, 3; over 20 and under 30, 30; over 30 and under 40, 25; over 40 and under 50, 15; over 50 and under 60, 9; over 60 and under 70, 6; over 70 and under 80, 7; over 80 and under 90, 1; stillborn, 10; unknown, 5. Total, 225.

The above table indicates a very healthy condition of the City; the gross mortality being at least 25 per cent less than for the month of January, 1864. This is probably owing to the coëxistence of two circumstances:—

The first is, that the past month (Jan., 1865) has been only moderately cold and dry, with no sudden and extreme atmospheric changes. The second is, that the river and atmosphere of the City have been much less offensive from putrid animal matter than during the corresponding month last year.

Chicago Medical College Commencement.—The Annual

Commencement exercises and conferring of Degrees, in Chicago Medical College, Medical Department of Lind University, will take place on the evening of the first Tuesday in March, 1865. The Valedictory Address will be delivered by Professor J. S. Jewell.

---

Transactions of the Amer. Med. Association for 1864. —This volume is now completed and ready for distribution. Our copy was received to-day, and will be duly noticed in the next number of the Examiner.

---

Illinois State Medical Society.—The following is the list of Committees expected to report at the Annual Meeting of the State Medical Society, to be held in Bloomington on the first Tuesday in May, 1865:—

*Committee on Practical Medicine and Epidemics:* J. Adams Allen, M.D., of Chicago; C. Goodbrake, M.D., of Clinton; H. Noble, M.D., of Hayworth.

*Committee on Surgery:* D. W. Young, M.D., of Aurora; A. L. McArthur, M.D., of Joliet; L. T. Hewins, M.D., of Oak Alla.

*Committee on Obstetrics:* W. H. Byford, M.D., of Chicago; Lucius Clark, M.D., of Rockford; S. T. Trowbridge, M.D., of Decatur.

*Commitiee on Drugs and Medicines:* John Bartlett, M.D., of Chicago; F. R. Payne, M.D., of Marshall; T. D. Fitch, M.D., of Chicago.

*Committee on Ophthalmology:* E. L. Holmes, M.D., of Chicago; R. E. McVey, M.D., of Waverly; J. S. Whitmire, M. D., of Metamora; F. B. Haller, M.D., of Vandalia.

*Committee on Spotted Fever:* J. S. Jewell, M.D., of Chicago.

*Committee on Orthopœdic Surgery:* David Prince, M.D., of Jacksonville.

The Committee of Arrangements and the Profession in Bloomington are preparing to give the Society a cordial reception, and we trust the coming meeting will be larger and more profitable than any that have preceded it. We hope the committees will have their reports fully prepared.

### AMERICAN MEDICAL ASSOCIATION.

The American Medical Association will hold its Sixteenth Annual Meeting in the City of Boston, on Tuesday, June 6th, 1865.

The following Committees are expected to report:—

On Insanity—Dr. H. R. Storer, Mass., Chairman.

On Exsection and its connection with Conservative Surgery—Dr. Lewis A. Sayres, N. Y., Chairman.

On Drainage and Sewerage of Large Cities, and their Influence on Public Health—Dr. W. J. C. Duhamel, D. C., Chairman.

On Alcohol and its Relations to Man—Dr. G. E. Morgan, Maryland, Chairman.

On Quarantine—Dr. Wilson Jewell, Penn., Chairman.

On Medical Ethics—Dr. John A. Murphy, Ohio, Chairman.

On the Microscope—Dr. James M. Corse, Pennsylvania, Chairman.

On the Relations which Electricity sustains to the Causes of Disease—Dr. Squire Littell, Penn., Chairman.

On the Morbid and Therapeutic Effects of Mental and Moral Influences—Dr. A. B. Palmer, Mich., Chairman.

On the Causes of the Extinction of the Aboriginal Races of America—Dr. Geo. V. Suckley, Md., Chairman.

On the Causes and Treatment of Ununited Fractures—Dr. Frank H. Hamilton, N. Y., Chairman.

On Diphtheria—Dr. Lucius Clark, Ill., Chairman.

On the Uses and Abuses of Pessaries—Dr. James P. White, N. Y., Chairman.

On International Medical Ethics—Dr. J. Baxter Upham, Mass., Chairman.

On Climatology and Epidemic Diseases—Dr. C. W. Parsons, R. I., Chairman.

On Autopsies in Relation to Medical Jurisprudence—Dr. T. C. Finnell, N. Y., Chairman.

On so-called Spotted Fever—Dr. James J. Levick, Penn., Chairman.

On the Introduction of Disease by Commerce, and the Means of its Prevention—Dr. A. Nelson Bell, N. Y., Chairman.

On Patent Rights and Medical Men—Dr. David Prince, Ill., Chairman.

On Revision of Plan of Organization—Dr. N. S. Davis, Chairman.

On Specialists—Dr. J. Homberger, N. Y., Chairman.

On Compulsory Vaccination—Dr. A. N. Bell, N. Y., Chairman.

On Rank of Medical Corps in the Army—Dr. C. S. Tripler, U. S. A., Chairman.

On Rank of Medical Corps in the Navy—Dr. T. L. Smith, N. Y., Chairman.

WM. B. ATKINSON, *Permanent Secretary.*

We insert the foregoing notice of the approaching meeting of the Association with much pleasure.

We anticipate such arrangements for the management of the several *Sections*, at the next meeting, as will secure the more thorough and interesting examination and discussion of all papers presented, and the restriction of entertainments, sight-seeing, &c., more nearly within their proper limits. It would be a good plan, therefore, if all who make reports or wish to present volunteer papers, would be ready on the first day, at the morning Session; that appropriate references may be made at once.

It is also extremely desirable that all reports and papers should be *fully prepared*, before presentation, that they may be passed directly from the Sections to the Committee of Publication, (if deemed worthy,) without being left, *perhaps months*, in the hands of their authors, for completion or revision. This would save the Committee of Publication much vexatious delay in the performance of their duties, and ensure a much earlier completion of the annual volume of Transactions. All we hear from the profession in the Middle and Western States, indicates a lively interest in the Meeting to be held in June next.

---

Arsenic-Eating in Styria—Dr. Craig Maclagan, in a paper lately read before the Edinburgh Medico-Chirurgical Society, shows that the "arsenic-eating" of Styria is no fable. On a late visit to Styria, he took occasion to investigate the matter himself; and, with his own eyes, saw one man swallow over four grains of arsenious acid at a dose, without suffering any poisonous symptoms. He afterwards found arsenic in the man's urine. Another man swallowed, in the doctor's presence, six grains of arsenious acid, eating it on bread and butter. "The man stated that he generally takes about the quantity we saw him swallow once a week, but with variations in the intervals, there being sometimes four days only, sometimes eight days, between the dose; that when he has a distance to walk to his work, he takes a larger dose, and is then in good spirits for about eight days; that if he, however, intermit it for fourteen days, he feels stiff in the feet, with general lassitude, and a craving for another dose. If his victuals are hard of digestion, he takes a dose to assist the stomach; and if he takes a rather full dose, he brings a good deal of wind off his stomach, but never vomits. He stated that his father had taken arsenic before him, and in considerable quantity; and that, in the immediate neighborhood of Liegist, numbers use it, several taking it daily, and many in larger doses than he. He said that all who take it are healthy; that he never knew of any one vomiting from its use; and he believed that, like the use of tobacco, if the dose is very gradually diminished, an arsenic-eater can break himself of the habit. —*British Med. Journal.*

---

TREATMENT OF ONYCHIA.—The disease of which Mr. de Moerloose, of Ghent, effects a cure in a few days by the application of nitrate of lead is not that which is popularly termed the growth of the nail into the flesh, but the fungous, ichorous, unhealthy ulcer, occurring especially in children at the root of the nails, which fall away, leaving a sore with swelled, jagged edges, causing a deformation of the finger or toe, and occasionally requiring amputation.

"Parents," says Mr. de Moerloose, "frequently convey their children to the hospital requesting me to cut off the diseased finger. This I have never consented to do, and I may add that I have always succeeded in effecting a rapid cure. A week or ten days in general, or at the farthest three or four weeks, have always been sufficient for the purpose, even under the most unfavorable circumstances. The remedy I resort to is the application, after the excision of any irregular horny excressences, of powdered nitrate of lead. The wound should be dressed once a day only; the pain promptly subsides, the swelling soon decreases, the secretion resumes a healthy character, and, in the course of five or six days, the sore is often thoroughly cleansed."

Mr. Wardrop, who refers the disease to syphilis, recommends mercury to be employed so as to affect the gums in about a fortnight (*a*); but although the affection may occasionally in the adult be traced to this cause, it is frequently met with in hospitals for infancy, where it appears more obviously connected with a scrofulous condition of the system, and much more rapidly yields to the very simple treatment above described than to any other medication.—*Med. and Surg. Rep.*

---

Respiration after Decapitation.—It appears that respiration to some extent can take place even after decapitation. A clever physiologist, M. Faivre, having some years ago made certain delicate experiments on the breathing of insects, came to the conclusion that in insects as well as in mammalia, the breathing faculty has its seat in some particular region of the nervous system, this region being the metathoracic or central ganglion. Another physiologist, M. Bandelot, a few days ago sent in a paper to the Academy of Science, in which he endeavored to show that M. Faivre was entirely wrong, and that the metathoracic ganglion was quite innocent of any action whatever in the breathing process. As to M. Bandelot's arguments it is feared that they can only be defended on the principle that the end jnstifies the means—for in order to ascertain this point in physiology, he tells us he took a caterpillar, the larva of a *libellula*, and summarily cut off its head; after which operation, the larva was good natured enough to continue breathing for twenty-four hours longer. Thus M. Bandelot had the satisfaction of ascertaining that the cerebral lobes have nothing to do with breathing, although some people are likely to object that the larva must have found it more inconvenient to breathe *with* a head than *without* one. But our author not being yet satisfied, took another larva, cut it right across through the middle, and discovered to his intense satisfaction, that the subject could breathe even without a metathoracic ganglion! Whether the larva was equally delighted is to be doubted, for M. Bandelot does admit there was some slight irregularity in the breathing.—*Med. and Surg. Rep.*

---

Treatment of Asthma.—It is almost impossible to lay down general rules applicable to the treatment of every case of asthma, because remedies efficacious in one instance prove injurious in another. We alluded in our last number to nitrated leaves and preparations of lobelia. The following is a prescription which Prof. Trousseau has often found serviceable when other remedies have failed:—

℞. Potassii iodidi, ℥ij.; Aquæ destil, ℥v.

The dose is one teaspoonful every day before dinner when dyspnœa persists after the cessation of the more violent paroxysms. This medicine facilitates expectoration and restores the freedom of respiration.—*Jour. Prac. Med. and Surg.*

---

Regeneration of Bone.—In a biographical sketch of Prof. J. R. Wood, N. Y., (in the *Med. & Surg. Rep.*) Dr. Francis states that Dr. Wood has reproduced almost every bone in the body by treating with profound respect the periosteum. His museum contains all the experiments on the human patient that were practiced on birds and quadrupeds by Duhamel and Flourens.

---

Obituary.—Wm. Senhouse Kirkes, M.D., died Dec. 8, 1864, of double pneumonia, with pericarditis, after an illness of five days. Dr. Kirkes was physician to St. Bartholomew's Hospital, and well known in this country as an author of an excellent hand-book on Physiology. His age was 41.

